# Symptomatic venous thromboembolism and mortality in orthopaedic surgery – an observational study of 45 968 consecutive procedures

**DOI:** 10.1186/1471-2474-14-177

**Published:** 2013-06-04

**Authors:** Lasse J Lapidus, Sari Ponzer, Hans Pettersson, Edin de Bri

**Affiliations:** 1 Section of Orthopaedics, Department of Clinical Science and Education, Södersjukhuset Karolinska Institutet, Stockholm, Sweden; 2 Section of Statistics, Department of Clinical Science and Education, Södersjukhuset Karolinska Institutet, Stockholm, Sweden

**Keywords:** Deep vein thrombosis, Mortality, Operation, Orthopaedic surgery, Prophylaxis, Pulmonary embolism, Thrombosis, Venous thromboembolism

## Abstract

**Background:**

Little information exists on the presentation of symptomatic venous thromboembolism (VTE) in orthopaedic surgery when a defined protocol for thromboprophylaxis is used. The objective with this study was to establish the VTE rate and mortality rate in orthopaedic surgery.

**Methods:**

We performed a prospective, single centre observational cohort study of 45 968 consecutive procedures in 36 388 patients over a 10 year period. Follow-up was successful in 99.3%. The primary study outcome was the incidence of symptomatic deep vein thrombosis (DVT), symptomatic pulmonary embolism (PE) and mortality at 6 weeks, specified for different surgical procedures. The secondary outcome was to describe the DVT distribution in proximal and distal veins and the proportion of VTEs diagnosed after hospital discharge. For validation purposes, a retrospective review of VTEs diagnosed 7–12 weeks postoperatively was also performed.

**Results:**

In total, 514 VTEs were diagnosed (1.1%; 95% CI: 1.10-1.14), the majority (84%) after hospital discharge (432 out of 514).With thromboprophylaxis, high incidence of VTE was found after internal fixation (IF) of pelvic fracture (12%; 95% CI: 5–26), knee replacement surgery (3.7%; 95% CI: 2.8-5.0), after internal fixation (IF) of proximal tibia fracture (3.8%; 95% CI: 2.3-6.3) and after IF of ankle fracture (3.6%; 95% CI: 2.9-4.4). Without thromboprophylaxis, high incidence of VTE was found after Achilles tendon repair (7.2%; 95% CI: 5.5-9.4). In total 1094 patients deceased (2.4%; 95% confidence interval (CI): 2.33- 2.44) within 6 weeks of surgery. Highest mortality was seen after lower limb amputation (16.3%, CI: 13.8-19.1) and after hip hemiarthroplasty due to hip fracture (9.6%, CI; 7.6-12.1).

**Conclusion:**

The overall incidence of VTE is low after orthopaedic surgery but our study highlights surgical procedures after which the risk for VTE remains high and improved thromboprophylaxis is needed.

## Background

Orthopaedic surgery is a well-known risk factor for venous thromboembolism (VTE), associated with short term mortality and long term morbidity [1]. The use of thromboprophylaxis is uncontroversial in high risk situations such as major joint surgery in hips and knees but less well defined, and sometimes questioned, for many other non-major orthopaedic procedures [2]. While a large number of reports have found a high incidence of VTE in screening studies, mostly asymptomatic, little information exists on the clinical presentation and the time-course of postoperative symptomatic VTE when a defined protocol for thromboprophylaxis has been used, particularly after lower limb fractures. Studies presenting the outcome of such protocols are particularly important when the daily clinical practice offers limited possibilities of postoperative VTE surveillance over the time at risk, leaving the VTE outcome of the patients unknown for the treating surgeon. The late occurrence of postoperative VTEs [3-5] and the widespread tradition with internist diagnosing and treating VTEs could also reduce the awareness of thromboembolic complications among orthopaedic surgeons.

This prospective study was performed in order to present the VTE outcome of a defined protocol for thromboprophylaxis and update the epidemiological data on VTE complications and mortality following a wide range of different orthopaedic procedures in large cohort of consecutive patients. The results represent all symptomatic events of VTE diagnosed within 6 weeks of surgery. Diagnostic investigations where performed only when the clinical suspicion of VTE was high.

### Methods

This prospective single center study was conducted at the Department of Orthopedics at Södersjukhuset Hospital in Stockholm between 1996 and 2005. The hospital is a public general hospital with a catchment area comprising about 600,000 inhabitants. All patients from the age of 15 operated at the department were included in the study, only patients participating in clinical trials involving thrombosis screening were excluded. A protocol for thromboprophylaxis was established at the department before study start, two minor changes were performed during the study period (Table 1). All patients operated at the department were assigned to a six week follow-up within the context of a clinical audit based on a questionnaire distributed to the patients in a prepaid envelope before discharge. In this questionnaire the patients were encouraged to report any adverse event after surgery and where this had been diagnosed and treated. One research nurse was responsible for the data collection (one and the same during the entire study period). In cases when the questionnaire was not returned (in approximately 50% of the cases), one or more reminders were sent to the patient. If necessary, contact with relatives or carers was established to ensure the follow-up. Medical records were requested for all (100%) adverse events reported in the questionnaires, also from care institutions elsewhere. An orthopaedic surgeon (5 different surgeons during the study period) reviewed the medical records in case of reported adverse event. All VTEs confirmed in medical records (patients diagnosed with and treated for VTE) and all deaths within 6 weeks of surgery were considered to be related to the surgery and thus included in the analysis. DVT was defined as any thrombosis diagnosed by venography or duplex sonography (CDS) and they were classified to be either distal (below the popliteal vein), proximal (in or above the popliteal vein) or located in the upper extremity. PE was diagnosed by computer tomography (CT), pulmonary angiography, ventilation-perfusion scintigraphy, or at autopsy. The images were reviewed in the clinical setting at the time of examination, not adjudicated separately in the study.

**Table 1:** Patient demographics, type of anaesthesia and thromboprophylaxis regimen in relation to the surgical procedure

	**Number of procedures**	**Median age**	**Female gender**	Type of anaesthesia*	**Thromboprophylaxis †**
	**(n = 45968)**	**(25th–75th percentile)**	**- n (%)**		
Hip replacement	4001	77.2 (68–84)	2794 (69.8)	>90% spinal	Yes ‡
(all indications)					
Knee replacement	1309	72.4 (64–78)	854 (65.2)	>90% spinal	Yes ‡
(all indications)					
Spine surgery	1320	50.4 (38–67)	677 (51.3)	>90% general	Yes
(all indications)					
Lower Limb Amputation	923	81.5 (74–87)	491 (53.2)	80-90% spinal	Yes
Pelvic and lower limb fractures	12739	78.7 (60–86)	8489 (66.6)	85-90% spinal	Yes
- IF (excl. foot fractures)					
Anterior Cruciate Ligament reconstruction	659	28.6 (23–34)	240 (36.4)	80-90% general	Yes
Knee arthroscopy	6057	38.6 (29–51)	2469 (40.8)	70-80% general	No
Achilles tendon rupture	764	39.8 (33–48)	131 (17.1)	70% local	No
Foot surgery	2373	52.8 (39–65)	1508 (63.5)	40% local	No
Upper extremity surgery	8695	55.0 (39–71)	5061 (58.2)	70% general	No
Miscellaneous procedures in the lower limb	1322	51.3 (34–71)	608 (46.0)	50% spinal	Individual §
Minor surgery	5806	59.9 (41–77)	3073 (52.9)	50% general	No

*IF* Internal fixation.

* Only the most frequent anaesthetic method is specified.

† Postoperative start with dalteparin 5000 U given subcutaneously once daily starting on the evening after surgery and continuing for 7 to 10 days.

For fracture patients’ preoperative thromboprophylaxis was used as follows;

Until March 31, 2000: dalteparin 5000 U subcutaneously at admission.

From April 1, 2000: 1000 mL RingerDextran60® intravenously at admission.

‡ Graduated compression stockings were used as a postoperative complement to LMWH until 9th of February 2000.

§ Prophylaxis based on surgeons preference. In general, prophylaxis was given after major procedures in bone (such as osteotomies) while no prophylaxis was given after soft tissue surgery.

All data concerning adverse events and surgical procedures were continuously collected and stored in a local database which was based on the unique Swedish personal identification number. A validation of the data base was performed in two retrospective analyses in order to verify correct registration of surgical procedures and VTE events within 6 weeks. For validation purposes, we also performed a retrospective review of VTE events diagnosed at the hospital 7–12 weeks after surgery (details are presented in the results section).

The primary study outcome was the incidence of symptomatic DVT and PE within six weeks of surgery. The secondary outcome was the distribution of DVTs in proximal and distal veins and the proportion of VTEs diagnosed after discharge from the hospital and the all-cause mortality at 6 weeks. The impact of two minor changes in the VTE prophylaxis protocol was evaluated (i.e. the VTE incidence after hip and knee replacement surgery was calculated with and without the use of postoperative compression stockings (used as a complement to LMWH until January 31st 2001) and the VTE incidence after internal fixation (IF) of lower extremity fractures (excluding foot fractures) was calculated before and after the change of preoperative prophylaxis from dalteparin to Ringer-Dextran60® (dextran 60) (April 1, 2000)).

The study was assessed by the Regional Ethics Committee in Stockholm (reference no. 296/03) but since this survey did not impact on the daily clinical practice, no formal approval was requested. However, the study was conducted according to ethical principles stated in the Declaration of Helsinki.

### Statistical analysis

Patient demographics were calculated as median age (with 25 and 75 percentile) because the age distribution was skewed at the time of the surgery (at their first surgical procedure if included more than once in the study). VTE incidence and mortality are presented with 95% confidence intervals (CI) using the Poisson distribution. Patients with more than one surgical procedure during a 90 day period were only included once in the analysis (the procedure with longest operating time was calculated). In the final analysis of VTEs, all events were counted until postoperative day 42, including DVTs and PEs primarily missed in the registration but detected in the validation process. The time to DVT or PE diagnosis is presented as the median number of days after surgery, differences were tested using Mann Whitney U test since censoring due to lot to follow-up was not a problem within the first 42 days. The impact of the changes in the VTE prophylaxis protocol was tested by the Fisher’s exact test. A p-value less than 0.05, two-tailed, were regarded as significant.

The statistical software used was IBM SPSS Statistics, Version 20 for Windows (IBM, New York, USA).

## Results

### Validation of surgical procedures and VTEs

The accuracy of the surgical procedure codes in our register was compared against the patient medical record database (Pasett®) for all cases coded as “IF of femur fracture” (NFJ) in the Swedish version of NCSP 96 (Nomesco Classification of Surgical Procedures) between the years 1999 and 2003 (n = 4586). All discrepancies were checked manually in patients’ records. There was a 99.9% (4578/4586) agreement in this coding between the different databases. One incorrect code was found in our register compared to seven incorrect codes in the reference database Pasett® (ie: IF when a hip prosthesis in fact had been performed).

To validate the completeness of our VTE registrations, we used Pasett® and identified all patients diagnosed with VTE at the hospital (1998–2005) and compared the findings with our database. In this analysis we found 14 more patients that had been treated for VTE (11 for DVT and 3 for PE) within 6 week after surgery. For one patient with a registered PE, the PE diagnosis had been re-evaluated and withdrawn at the treating department although this had not passed through to our register. Corrections were subsequently performed in the register before analysis.

To validate the methodology, using a 6 week monitoring of postoperative VTE, we used Pasett® and identified all patients diagnosed with VTE at the hospital 7 to 12 weeks after surgery. In this analysis we found another 37 DVTs (7 after proximal femoral fracture, 7 after hip replacement, 5 after knee replacement, 5 after Achilles tendon repair, 3 after ankle fracture, 3 after other lower extremity surgery, 2 after femur fracture, 2 after upper extremity surgery, 1 after foot surgery, 1 after minor surgery and 1 after lower limb amputation) and 9 PEs ( 5 after proximal femoral fracture, 1 after hip replacement, 1 after knee replacement, 1 after foot surgery and 1 after knee arthroscopy). These late VTEs represented 10% (46/473) of postoperative VTEs during the years 1998–2005.

### Patient characteristics

A total of 46 796 surgical procedures were performed during the study period and enrolled in our clinical audit database, 530 procedures were excluded from the analysis (93 procedures on infants and 427 procedures on patients participating in clinical trials involving thrombosis screening) and 308 cases (0.7%) were lost to follow-up leaving 45 968 procedures in 36 388 patients to be included in the analysis, 56.6% of patients being female (20 590). The median age was 71.1 years (25 and 75 percentile: 51–83 years) for women and 50.4 years (25 and 75 percentile: 34–70 years) for men. The median age for patients diagnosed with a VTE was 74.2 years (25 and 75 percentile: 56–83 years) for women and 54.5 years (25 and 75 percentile: 37–70 years) for men. Median age and gender distribution in relation to different surgical procedures is presented in Table 1, 2 and 3.

**Table 2:** Age, gender, VTE incidence and all-cause mortality following hip arthroplasty in relation to indication for surgery

**Type of surgery**	**Number of procedures (n = 4001)**	**Median age- (25th-75th percentile)**	**Female gender - n (%)**	**Mortality – n (%)**	**All VTE – n (%; 95% CI)**	**PE – n (%; 95% CI)**	**DVT – n (%; 95% CI)**
**Total hip replacement**							
-Degenerative hip disorder	1691	71.1 (62–78)	1101 (65.1)	10 (0.6; 0.3-1.1)	40 (2.4; 1.7-3.2)	14 (0.8; 0.5-1.4)	28 (1.7; 1.1-2.4)
**-**Fracture	348	78.5 (73–83)	272 (78.2)	9 (2.6; 1.3-5.0)	10 (2.9; 1.5-5.3)	3 (0.9; 0.3-2.7)	7 (2.0; 1.0-4.2)
**-** Fracture sequelae	522	77.9 (70–84)	392 (75.1)	13 (2.5; 1.4-4.3)	14 (2.7; 1.6-4.5)	6 (1.2; 0.5-2.6)	8 (1.5; 0.8-3.1)
**Hemiarthroplasty**							
- hip fracture	743	84.9 (80–89)	565 (76.0)	71 (9.6; 7.6-12.1)	7 (0.9; 0.4-2.0)	6 (0.8; 0.4-1.8)	1 (0.1; 0.02-1.0)
- Fracture sequelae	239	84.9 (81–90)	192 (80.3)	15 (6.3; 3.8-10.4)	3 (1.3; 0.4-3.9)	0	3 (1.3; 0.4-3.9)
**Hip prosthesis revision**	429	74.8 (64–82)	251 (58.5)	3 (0.7; 0.2-2.2)	5 (1.2; 0.5-2.8)	0	5 (1.2; 0.5-2.8)
**Hip replacement** - malignancy	29	64.3 (58–78)	21 (72.4)	1 (3.4; 0.5-24.5)	1 (3.4; 0.5-24.5)	0	1 (3.4; 0.5-24.5)

Thromboprophylaxis was used for 7 to 10 days.

Fracture sequelae = osteonecrosis of the femoral head or non-union and or implant failure after hip fracture surgery.

**Table 3:** Age, gender, VTE incidence and all-cause mortality following internal fixation (IF) of lower extremity fracture

**Fracture location**	**Number of procedures (n = 12739)**	**Median age- (25th-75th percentile)**	**Female gender – n (%)**	**Mortality – n (%; 95% CI)**	**All VTE – n (%; )%% CI)**	**PE – n (%; 95% CI)**	**DVT – n (%; 95% CI)**
Pelvis	52	46 (33–57)	21 (40)	1 (2; 0–14)	6 (12; 5–26)	3 (6; 2–18)	3 (6; 2–18)
Proximal femur	8402	83.3 (77–88)	6129 (72.9)	617 (7.3; 6.8-7.9)	96 (1.1; 0.9-1.4)	39 (0.5; 0.3-0.6)	57 (0.7; 0.5-0.9)
Femur, other	581	78.4 (62–86)	433 (74.5)	27 (4.6; 3.2-6.8)	12 (2.1; 1.2-3.6)	2 (0.3; 0.1-1.4)	10 (1.7; 0.9-3.2)
Patella	169	62.3 (45–74)	95 (56.2)	0	0	0	0
Tibia, proximal	395	52.6 (39–66)	208 (52.7)	2 (0.5; 0.1-2.0)	15 (3.8; 2.3-6.3)	3 (0.8; 0.2-2.4)	13 (3.3; 1.9-5.7)
Tibia, diaphysis	502	46.6 (33–58)	194 (38.6)	3 (0.6; 0.2-1.9)	11 (2.2; 1.2-4.0)	3 (0.6; 0.2-1.9)	8 (1.6; 0.8-3.2)
Tibia, distal	175	47.6 (37–61)	77 (44.0)	0	3 (1.7; 0.6-5.3)	2 (1.1; 0.3-4.6)	1 (0.6; 0.1-4.1)
Ankle	2463	51.3 (37–62)	1332 (54.1)	5 (0.2; 0.1-0.5)	89 (3.6; 2.9-4.4)	11 (0.4; 0.2-0.8)	78 (3.2; 2.5-4.0)

Thromboprophylaxis was used for 7 to 10 days.

### Surgical characteristics

Surgical procedures, thromboprophylaxis and anaesthetic method are presented in Table 1. The indication for hip prosthesis surgery is specified separately in Table 2 and the different lower extremity fractures are presented in detail in Table 3.

The indication for knee replacement surgery was osteoarthritis or rheumatoid arthritis (n = 1226), knee revision surgery (n = 81) and fracture (n = 2). Spinal surgery was performed due to degenerative spine disorders (n = 1135), fracture (n = 95), malignancy (n = 70) or infection (n = 20). Amputation of the lower limb was performed due to compromised circulation or infection in 912 cases and due to trauma in 11 cases. The amputation level was trans-tibial (n = 546), a disarticulation of the knee (n = 244), trans-femoral (n = 127), a disarticulation of the hip (n = 5) and a disarticulation of the talo-crural joint in one case. Miscellaneous procedures in the lower extremity include different skeletal and soft tissue procedures (such as different correction osteotomies, patella tendon repairs and quadriceps tendon repairs). Minor surgical procedures represent mostly wound debridements, closed reductions of joint dislocations and other related procedures.

### Characteristics of VTEs

In total, 401 DVTs (0.9%) and 123 PEs (0.3%) were diagnosed during the study period. In ten patients DVT and PE were diagnosed simultaneously. A proximal DVT was found in 133 (33%) cases, a distal DVT in 241 (60%) cases and an arm thrombosis was found in 2 cases (including one patient with fatal PE). In 25 cases (6%) the distribution of the lower extremity DVT was not specified.

The median time for DVT diagnosis for all patients was 16 days (range 0–42) after surgery and 342 out of 401 (85%) DVTs were diagnosed after hospital discharge. The median time for DVT diagnosis differed significantly (p = < 0.001) after hip replacement (26 days, range 0–42) compared to knee replacements (6 days, range 0–42). Consequently the majority (92%) of DVTs after hip replacement was diagnosed after hospital discharge (49 out of 53) compared to 45% (26 out of 47) after knee replacements. The distribution of DVTs in distal and proximal veins is presented in Table 4. For fracture patients treated with IF the share of proximal DVT was 53% (n = 31) for pelvis and femur fractures combined, 20% (n = 5) for tibia fractures and 16% (n = 12) for ankle fractures.

**Table 4:** VTE incidence and all-cause mortality in relation to the surgical procedure

	**Mortality**	**All VTE**	**PE**	**DVT**	**Rate of proximal DVT -n (%) †**	**Rate of post-discharge VTE – n (%)**
	**(n = 1094) – n (%)**	**(n = 513) –n (%; 95% CI)**	**(n = 123)– n (%; 95% CI)**	**(n = 399) – n (%; 95% CI)**		
Hip replacement*	122 (3.0; 2.6-3.6)	80 (2.0; 1.6-2.5)	29 (0.7; 0.5-1.0)	53 (1.3; 1.0-1.7)	33 (70)	72 (90)
(all indications)						
Knee replacement*	1 (0.1; 0.01-0.5)	49 (3.7; 2.8-5.0)	3 (0.2; 0.1-0.7)	47 (3.6; 2.7-4.8)	14 (30)	25 (57)
(all indications)						
Spine surgery (all indications)*	8 (0.6; 0.3-1.2)	4 (0.3; 0.1-0.8)	3 (0.2; 0.1-0.7)	1 (0.1; 0.01-0.5)	1 (100)	3 (75)
Lower Limb Amputation*	150 (16.3; 13.8-19.1)	8 (0.9; 0.4-1.7)	7 (0.8; 0.4-1.6)	2 (0.2; 0.05-0.9)	0	3 (38)
Pelvic and lower limb fractures - IF	655 (5.1; 4.8-5.6)	232 (1.8; 1.6-2.1)	63 (0.5; 0.4-0.6)	170 (1.3; 1.1-1.6)	48 (31)‡	199 (86) §
(excl. foot fractures)						
Knee arthroscopy	4 (0.1; 0.02-0.2)	28 (0.5; 0.3-0.7)	3 (0.05; 0.02-0.2)	28 (0.5; 0.3-0.7)	14 (52)	24 (83)
Anterior Cruciate Ligament reconstruction*	0	3 (0.5; 0.1-1.4)	0	3 (0.5; 0.1-1.4)	1 (33)	3 (100)
Achilles tendon ruptures	0	55 (7.2; 5.5-9.4)	0	55 (7.2; 5.5-9.4)	8 (15)	55 (100)
Foot surgery	1 (0.04; <0.01-0.3)	16 (0.7; 0.4-1.1)	1 (0.04; <0.01-0.3)	15 (0.6; 0.4-1.0)	0	15 (94)
Upper extremity surgery	34 (0.4; 0.3-0.5)	15 (0.2; 0.1-0.3)	8 (0.1; 0.05-0.2)	7 (0.1; 0.04-0.2)	5 (83)	12 (86)
Miscellaneous procedures in the lower limb	26 (2.0; 1.3-2.9)	19 (1.4; 0.9-2.2)	4 (0.3; 0.1-0.8)	17 (1.3; 0.8-2.1)	8 (47)	15 (79)
Minor surgery	93 (1.6; 1.3-2.0)	5 (0.1; 0.04-0.2)	2 (0.03; 0.01-0.1)	3 (0.1; 0.02-0.2)	1 (33)	3 (60)

*IF* Internal fixation.

* Thromboprophylaxis given according to protocol.

† Based on 374 DVTs with defined distribution of the DVTs.

‡ Rate of proximal DVT was 53% (n = 31) for pelvis and femur fractures, 20% (n = 5) for tibia fractures and 16% (n = 12) for ankle fractures.

§ Rate of post-discharge VTE was 85% (n = 97) for pelvis and femur fractures, 72% (n = 21) for tibia fractures and 91% (n = 81) for ankle fractures.

In total, 38 out of 123 PEs were fatal (31%), 20 of these were diagnosed at autopsy and the other 18 were diagnosed with CT or ventilation-perfusion scintigraphy before death.

Fatal events of PE occurred after IF of femur fracture (n = 18), lower limb amputation (n = 5), minor procedures (n = 1), IF of tibia fracture (n = 1), IF of ankle fracture (n = 1), spine surgery (n = 1), upper extremity surgery (n = 1) and hip replacement (n = 10, distributed to following indications: fracture (n = 4), fracture sequele (n = 3) and degenerative hip disorder (n = 3)).

The median time to PE diagnosis was 23 days (range 0–42) and 99 out of 123 (80%) of PEs were diagnosed after hospital discharge.

### VTE incidence in relation to different surgical procedures

The VTE incidence after different surgical procedures is presented in Tables 4, 2, 3. In Table 2 the VTE incidence after hip replacement is presented in relation to the indication for surgery and in Table 3 the VTE incidence after IF of lower limb fractures is presented. Some results are noteworthy. With the use of thromboprophylaxis, the highest incidence of VTE was found after IF of pelvic fractures (12%; 95% CI: 5–26), knee replacement surgery (3.7%; 95% CI: 2.8-5.0), after IF of proximal tibia fractures (3.8%; 95% CI: 2.3-6.3) and after IF of ankle fractures (3.6%; 95% CI: 2.9-4.4).

Without thromboprophylaxis, the highest incidence of VTE was found after repair of Achilles tendon ruptures (7.2%; 95% CI: 5.5-9.4).

No significant difference (p = 0.6) in VTE incidence was found when changing the preoperative thromboprophylaxis for lower extremity fracture patients (13 830 patients included in the analysis) from dalteparin to RingerDextran60® (1.7% and 1.9%, respectively). Nor did we find any significant difference in VTE incidence after hip and knee replacement surgery in 5310 patients when we stopped using postoperative compression stockings, the VTE incidence was 2.7% before and 2.3% after the change (p = 0.4).

### All-cause mortality

The overall mortality at 6 weeks was 2.4% (n = 1094) with a median of 14 days (SD 12) to death. Autopsy was performed in 45 (4%) cases. In 20 out of 45 cases a PE was found to have either contributed to death or to have been the major cause of death (as described by the pathologist). The mortality in the different groups is presented in Tables 4, 2, 3. The highest 6 week mortality was seen after lower limb amputation (16.3%; 95% CI: 13.8-19.1), after hip hemiarthroplasty due to hip fracture (9.6%; 95% CI: 7.6-12.1) and after IF of proximal femoral factures (7.3%; 95% CI: 6.8-7.9). These patients also had the highest median ages in the study population; 82 years, 85 years and 83 years, respectively.

The 6 week mortality after hip fracture surgery (IF and hip replacement combined) was 1.8% (2/110) in patients with a confirmed diagnosis of DVT and 6.0% (737/12289) in patients without a DVT diagnosis (p = 0.07).

## Discussion

The present prospective observational study provides a comprehensive analysis of the epidemiology of VTE after orthopaedic surgery in patients enrolled in a well-defined protocol for thromboprophylaxis. We demonstrate how mortality and the outcome of VTE events differs between procedures, that the proportion of VTEs diagnosed after discharge from the hospital is correlated to the type of surgery as well as the distribution of DVTs in proximal and distal veins. We also show that no additional protection against VTE was found by using compression stockings after major hip and knee surgery.

Previous epidemiological studies in elective hip and knee replacement surgery have shown rates of symptomatic VTE between 1.1 and 10.6% [6-10]. Our results is similar to these, with a VTE rate of 2.4% (95% CI: 1.7-3.2) after hip replacement (degenerative hip disorder) and 3.7% (95% CI: 2.8-5.0) after knee replacement, but in the higher range compared to the 1.3% after hip replacement and 2.8% after knee replacement in the FOTO-study [11], particularly when considering the shorter follow-up in our study (42 days *vs*. 90 days). The difference could be explained by the shorter duration of thromboprophylaxis in our study (7–10 days *vs*. 36 days), a difference that has a significant impact on the risk of developing postoperative VTE, at least after hip replacement surgery [12-16]. Our findings, with a significantly lower rate of VTE after hemiarthoplasty of the hip (0.9%; 95% CI: 0.4-2.0) and (1.3%; 95% CI: 0.4-3.9) for different indications compared to that after total hip replacement for different indications (2.4%; 95% CI: 1.7-3.2), (2.7%; CI: 1.6-4.5), (2.9%; 95% CI: 1.5-5.3) is surprising and could represent a positive effect of less traumatizing surgery in hemiarthroplasties. However, it is more likely that VTEs in these more elderly patients in a higher degree remains undiagnosed and possibly more often result in sudden death rather than in the diagnosis of a VTE, reflected by the high mortality. This could be supported by the fact that PEs were found more often than DVTs after hip hemiarthroplasty and by the trend with reduced 6 week mortality after hip surgery (IF and hip replacement) in patients with a confirmed VTE diagnosis compared to those without, 1.8% (2/110) and 6.0% (737/12289), respectively (p = 0.07). Poor clinical awareness of thromboembolic complications or a high rate of undiagnosed fatal PEs, possibly due to asymptomatic DVTs, could also explain the low rate of VTE after IF of proximal femur fractures, 1.1% (95% CI: 0.9-1.4) and after lower limb amputations, 0.9% (95% CI: 0.4-1.7). These results are remarkable considering that VTE is the fourth most common cause of death, contributing to 14% of all deaths after hip fracture surgery [17,18], and also represent a significant risk factor for deaths after lower limb amputation [19]. The VTE rates after IF of other femur fractures (2.1%; 95% CI: 1.2-3.6) could be biased by the same reason. An unacceptably high VTE incidence despite thromboprophylaxis was found after IF of lower limb fractures below the knee (excl. foot fractures) and better prophylactic regimes are required in these injuries. Proximal tibia fractures seem to be correlated with a particularly high risk for VTE compared to other tibia fractures [20,21] however this difference was not significant in our study. The rate of VTE after ankle fracture (3.6%; 95% CI: 2.9-4.4) was significantly higher than the reported 0.4% found in one analysis of over 45 000 cases [2], a difference that could be explained by insufficient data quality in the hospital admission database used in the later study. In one placebo-controlled randomized trial (RCT), we were unable to significantly reduce the risk for VTE after ankle fracture surgery when extending the prophylaxis with dalteparin from one to six weeks [22]. We found similar results, with no significant risk reduction of VTE, in a second placebo-controlled RCT after Achilles tendon repair [23]. In the present study, the DVT rate was as high as 7.2% (CI: 5.5-9.4). The outcome of VTEs after Achilles tendon rupture seems to be favorable however, since long term follow-up studies have not shown any evidence of post thrombotic syndrome (PTS), neither after asymptomatic DVT [24] nor after symptomatic DVT [25]. This is probably explained by the high (85%) rate of distally distributed DVTs found in the present study. Contrary to our findings, significant reduction of VTE in plaster cast immobilization of ankle fractures and soft-tissue injuries using low molecular weight heparin (LMWH) was shown in a more recent meta-analysis [26].

The finding of only 4 VTEs in 1320 spine procedures in our study (0.3%, CI: 0.1-0.8) is significantly lower than the reported rate of DVT and PE (3.7% and 2.2%, respectively) in a review of lumbar fusion surgery without thromboprophylaxis [27]. The difference in results could be explained by the fact that our protocol recommended thromboprophylaxis after procedures on the spine. A low rate of VTE was also found in a recent study when mechanical prophylaxis was used [28].

Low VTE incidence in procedures performed without routine thromboprophylaxis was found after knee arthroscopy (0.5%; 95% CI: 0.3-0.7), foot surgery (0.7%; 95% CI: 0.4-1.1) and upper extremity surgery (0.2%; 95% CI: 0.1-0.3) and therefore routine thromboprophylaxis does not seem justified in these procedures. These results are comparable with previous reports in the topic [29-31].

The distribution of DVTs in proximal and distal veins followed the fracture type with higher rate of proximal DVTs in proximal fractures and vice versa. This relation was found also after hip and knee replacement, also demonstrated in a previous study [4]. The higher rate of proximal DVTs after hip replacement has been assigned to a local injury to the femoral vein occurring when the leg is flexed and rotated during the surgery [32-34], it is likely that local vascular injuries also play an important role in the formation of DVT after lower limb fracture.

The rate of DVTs diagnosed after hospital discharge was 92% after hip replacement and 55% after knee replacement (for VTEs corresponding rates were 90% and 57%, respectively). Similar differences have been reported previously [3,29]. Reasonable mechanisms for late-occurring VTE have been related to prolonged activation of the coagulation system [35,36]. A prolonged reduction in venous outflow has also been described, persisting for 6 weeks after hip replacement [37,38] but normalizing during the first week after knee replacement [39], differences that possibly could be explained by the use of tourniquet and the associated venous stasis and more extensive soft-tissue damage in knee replacement surgery, leading to extensive local release of tissue factor [12]. These findings support the present guidelines recommending prolonged prophylaxis, especially important after hip replacement surgery [1].

The results with 513 VTEs diagnosed over a 10 year period correspond in average to one event per week on a department with more than 30 orthopedic surgeons. This indicates that the diagnosis of a postoperative VTE is an uncommon finding for a single orthopedic surgeon. Since most of the VTEs were diagnosed after discharge from hospital, often by other physicians than orthopedic surgeons, a systematic feedback or registration of postoperative VTE could be important for complication awareness and quality control after surgery. With this study, we demonstrate that registration of adverse events can be performed with high validity when using dedicated personnel. A number of VTEs remained however unregistered, the reason for this could not be identified but these cases where consequently included retrospectively in the analysis. Another possible source of underestimation of the true rate of VTE could be poor clinical awareness of thromboembolic complications as well as difficulties in distinguishing VTE symptoms from normal postoperative findings. In addition, many patients with a fatal PE remained most certainly undiagnosed in our study due to a low autopsy rate and since the course of this condition is often very rapid and results in sudden death before resuscitation [40,41]. The extremely high rate of fatal PE (20 out of 45) seen in the autopsies could possibly be explained by selection bias but the results still indicates that fatal PE remains a serious threat after orthopaedic surgery. Based on the validity control, we believe that the overall data quality in our study is highly valid. Furthermore, the annual VTE and mortality incidence has shown a consistency over the ten year study period (data not shown) not affected by other possible changes in routines at the department over time. The relatively short postoperative follow-up period of 6 weeks is a limitation of our study. It is known that the risk for VTE after major orthopaedic surgery remains higher than normal for 2–3 months [3-5]. The majority of thromboembolic events occur however, during the first post-surgical month [4]. This is confirmed in our retrospective analysis with only 10% late occurring VTEs in postoperative week 7 to 12, findings that also are presented. We therefore believe that our results provide the reader valuable information regarding postoperative VTE after orthopaedic surgery. Strengths of the study include a remarkable follow-up rate of a large number of consecutive patients in a prospective study including VTEs also diagnosed elsewhere.

## Conclusions

Although the overall incidence of VTE after orthopaedic surgery was low our study highlights surgical procedures after which the risk for VTE remains high and improved thromboprophylaxis is needed. Since most of the VTEs present after hospital discharge special attention is needed to prevent and diagnose these late thromboembolic events.

## Competing interests

Lasse J Lapidus has received honorarium as a member of the VTE advisory board at Bayer AB and Boeringer Ingelheim AB. No financial, intellectual or other contribution has been received for the present study.

Karolinska Institutet, Department of Clinical Science and Education at Södersjukhuset provided grants to fulfil this study.

## Authors’ contributions

LJL and EdB orginated the idea for the study, contributed to its design with SP and HP. Statistical analysis was performed by HP and LJL. LJL collected and validated data and prepared the manuscript. All authors have read, edited and approved the final manuscript.

## Pre-publication history

The pre-publication history for this paper can be accessed here:

http://www.biomedcentral.com/1471-2474/14/177/prepub
